# Maximizing H_2_ Production from a Combination of Catalytic Partial Oxidation of CH_4_ and Water Gas Shift Reaction

**DOI:** 10.3390/molecules30020271

**Published:** 2025-01-11

**Authors:** Pannipa Tepamatr, Pattarapon Rungsri, Pornlada Daorattanachai, Navadol Laosiripojana

**Affiliations:** 1 Department of Chemistry, Faculty of Science and Technology, Thammasat University, Pathumthani 12120, Thailand; pannipa@tu.ac.th; 2 The Joint Graduate School of Energy and Environment, CHE Center for Energy Technology and Environment, King Mongkut’s University of Technology Thonburi, 126 Pracha Uthit Rd., Bang Mod, Thung Khru, Bangkok 10140, Thailand

**Keywords:** partial oxidation of methane, water gas shift, Gd, dual-bed catalyst

## Abstract

A single-bed and dual-bed catalyst system was studied to maximize H_2_ production from the combination of partial oxidation of CH_4_ and water gas shift reaction. In addition, the different types of catalysts, including Ni, Cu, Ni-Re, and Cu-Re supported on gadolinium-doped ceria (GDC) were investigated under different operating conditions of temperature (400–650 °C). Over Ni-based catalysts, methane can easily dissociate on a Ni surface to give hydrogen and carbon species. Then, carbon species react with lattice oxygen of ceria-based material to form CO. The addition of Re to Ni/GDC enhances CH_4_ dissociation on the Ni surface and increases oxygen storage capacity in the catalyst, thus promoting carbon elimination. In addition, the results showed that a dual-bed catalyst system exhibited catalytic activity better than a single-bed catalyst system. The dual-bed catalyst system, by the combination of 1%Re4%Ni/GDC as a partial oxidation catalyst and 1%Re4%Cu/GDC as a water gas shift catalyst, provided the highest CH_4_ conversion and H_2_ yield. An addition of Re onto Ni/GDC and Cu/GDC caused an increase in catalytic performance because Re addition could improve the catalyst reducibility and increase metal surface area, as more of their surface active sites are exposed to reactants.

## 1. Introduction

H_2_ is one of the most important industrial chemicals because it is a clean fuel and has an absence of undesirable combustion products. Therefore, its application as a transportation fuel is the greatest incentive. H_2_ demand in oil refining and coal processing industries is increasing [1,2,3]. The largest quantities of hydrogen are currently produced by steam reforming of CH_4,_ but this process is highly endothermic. The catalytic partial oxidation of CH_4_ (CPOM) is a potential alternative to the steam reforming processes. This process produces the synthesis gas through the reaction of methane with oxygen. The CPOM is an exothermic reaction that requires lower energy input and capital cost in comparison with steam reforming. Generally, partial oxidation of methane (POM) is performed in the presence of heterogeneous catalysts [4,5]. The noble metal-based catalysts (such as Pt, Pd, Rh, and Ru) are considered the most active catalysts for POM, but they exhibit high cost and low abundance. Therefore, those limitations greatly hamper their large-scale applications [5,6]. Ni catalysts are widely used in many industrial processes because nickel shows good catalytic activity and low cost. Nevertheless, Ni gets easily deactivated due to coke deposition and particle sintering. In order to avoid these problems, the addition of suitable promoters is a common strategy to promote catalytic activity and stability of Ni catalysts [7,8]. Ceria and doped ceria are widely utilized as supports to promote catalytic activity and reduce coke formation in many reactions, such as oxidation and reforming reactions, because of their high oxygen storage capacity and redox activity [9,10,11]. In particular, the presence of gadolinium-doped ceria (GDC) reduced the amount of carbon deposition on the catalyst surface. It was found that the amount of carbon deposited on gadolinium-doped ceria (GDC) was observed to be much lower than on ceria support because of the higher oxide ion conductivity introduced by doping [11].

In general, water gas shift (WGS) is applied to adjust the proportions of the resultant product gases for further applications [12,13,14,15]. From our previous work, we have studied the WGS reaction to increase the H_2_/CO ratio by using Cu on CeO_2_ and GDC as catalysts. The results demonstrated that 5%Cu/GDC showed the best catalytic performance of 80% CO conversion at 400 °C. Moreover, the experimental results revealed that an addition of Re promoter to Cu greatly improved the catalytic activity of Cu catalysts [14]. 

The two-stage reaction is a more appropriate route for H_2_ production than the one-stage reaction because of more flexible operation and higher H_2_ production efficiency [16,17,18,19]. A dual-bed system (Pt/Ni catalyst) was compared to single monolith platinum and rhodium catalysts for the synthesis gas production from the auto-thermal partial oxidation of methane [16]. They found that a dual-bed system of Pt/Ni catalyst performed as well as the Rh catalyst, but the price costs of the Rh catalyst, including blank monolith, are higher than that of the Pt/Ni catalyst by about 40%. In addition, Batista and co-workers studied the simultaneous steam reforming of ethanol and water gas shift reactions on single and double-bed reactors containing Co/SiO_2_ and Fe_2_O_3_/Cr_2_O_3_ as catalysts [17]. The results showed that the CH_4_ and CO concentrations generated from the double bed reactor were lower than those on pure Co/SiO_2_ catalysts.

The present work extends this technology to generate additional hydrogen by a combination of the catalytic partial oxidation of CH_4_ and water gas shift reaction (WGSR). Furthermore, non-noble metal-based catalysts were used in this work to reduce the prices and costs of the catalyst. The CPOM generates H_2_ in the first reactor and then follows by the water gas shift reaction to produce additional H_2_ by allowing the syngas to react with steam in a second reactor. Due to the exothermic nature of the WGSR, the reaction is conducted at lower temperatures to increase the production of H_2_ from CO. However, higher temperatures are required to achieve the necessary reaction kinetics. These higher temperatures are still crucially lower than those used for the CPOM [20]. Thus, a combination of two reactors is essential for maximizing H_2_ generation. This research paper presented the hydrogen production system on a single-bed and a dual-bed catalyst system. The performance of different hydrogen production systems with different types of catalysts, including Ni/GDC, Cu/GDC, Ni-Re/GDC, and Cu-Re/GDC were investigated under different operating conditions of temperature. Moreover, characterization techniques were performed to determine the structural/textural properties of the synthesized catalysts.

## 2. Experimental

### 2.1. Catalyst Preparation and Characterization

In this work, all catalysts were prepared by the impregnation method; 10%Gadolia-doped ceria (GDC, Daiichi, Japan) was selected as the catalyst support. Prior to catalyst preparation, GDC was crushed and sieved to obtain a particle size of about 0.425 mm. Then, GDC was impregnated with metal salt solutions of nickel (II) nitrate hexahydrate ((Ni(NO_3_)_2_⋅6H_2_O, Univar), copper nitrate trihydrate (Cu(NO_3_)_2_·3H_2_O, Sigma-Aldrich, Almelo, The Netherlands), and ammonium perrhenate (H_4_NO_4_Re, Sigma-Aldrich). The amount of total metal loading was fixed as 5 wt. % for both the monometallic and bimetallic catalysts; 5%Ni, 5%Cu, 1%Re-4%Ni, and 1%Re-4%Cu were prepared for use in the single-bed and dual-bed catalyst systems. After the impregnation step, all materials were dried overnight in an oven at 105 °C and calcined at 650 °C in air for 6 h.

X-ray powder diffraction of Ni and Cu catalysts was performed by X’Pert Pro diffractometer (PANalytical, Almelo, The Netherlands) with a current of 40 mA and 40 kV. Nickel-filtered Cu Kα radiation was used to collect the X-ray diffractograms over a 2θ range of 20–80. The CeO_2_ crystallite size was calculated from the full width at half maximum of the strongest (111) reflection using Scherrer’s equation. 

The specific surface area, pore diameter, and pore volume of catalysts were measured by N_2_ adsorption–desorption isotherms at 77 K using the BELSORP-MAX instrument (MicrotracBEL, Osaka, Japan) by using nitrogen adsorption–desorption techniques. The catalyst samples were degassed at 120 °C for 4 h and measured for adsorption at −196 °C.

A field emission scanning electron microscope (SU-8030, Hitachi High-Technologies Corporation, Tokyo, Japan) was operated in conjunction with energy dispersive X-ray spectroscopy (EDX) for the elemental analysis using secondary electrons and an acceleration of 30 kV.

H_2_-temperature-programmed reduction (H_2_-TPR) was carried out by using a BELCAT-B instrument (MicrotracBEL, Osaka, Japan) with 50 mg of the sample. Prior to the experiments, the samples were pre-treated at 120 °C for 30 min in He. The reduction was conducted in a 5 vol.% H_2_ in Ar at a heating rate of 10 °C/min from 40 to 1000 °C. The hydrogen consumption was analyzed using a thermal conductivity detector (TCD, MicrotracBEL, Osaka, Japan). 

A Perkin Elmer System 2000 FTIR/FT-Raman (Perkin Elmer, Rodgau, Germany) was performed under Ar ion laser irradiation at a wavelength of 532 nm and 10 mW maximum power. The Raman spectra were collected over the range of 300–1000 cm^−1^ using an operating spectra resolution of 1.0 cm^−1^.

Metal surface area and dispersion were measured from the total gas chemisorption using a BELCAT-B instrument. Prior to the measurement, samples were reduced with H_2_ at 400 °C for an hour and then cooled to 50 °C under a flow of He. After cooling, the CO chemisorption pulse was analyzed using 10%CO/He with the rate of 30 mL min^−1^ at 50 °C. The flow of CO out from the reactor was monitored by a thermal conductivity detector.

Perkin Elmer TGA/DTA 6300 instrument (Perkin Elmer, Rodgau, Germany) was used for the measurement of the weight loss in Ni catalysts as a function of temperatures up to 800 °C with a heating rate of 20 °C/min. 

The chemical composition of the catalyst surface was determined by an X-ray photoelectron spectrometer (XPS, AXIS ULTRA DLD, Kratos analytical, Manchester, UK). Before the measurement, the catalysts were reduced under a flow of 5%H_2_/N_2_ at 400 C for 1 h. X-ray anodes ran at 15 kV, 10 mA, and 150 W. The photoelectrons were detected with a hemispherical analyzer positioned at an angle of 90 with respect to the normal sample surface. The spectra were calibrated using the C1s line.

### 2.2. Apparatus and Procedures

The hydrogen generation system was carried out in a fixed-bed tubular quartz reactor (OD 3/8 in, length 400 mm). The supplied gas section consisted of high-purity methane (CH_4_, UHP grade 99.999%) and oxygen (O_2_, UHP grade 99.995%) as reactants and argon (Ar, UHP grade 99.995%) as a carrier gas. The mass flow controllers were used to precisely control gas flow into the reactor. In order to test the performance of catalysts and hydrogen production systems, 0.1 g of catalyst samples were placed in the reactor. For packing catalysts in the case of a dual-bed catalyst system, catalysts for the POM were the first bed, and catalysts for the WGS reaction were the second bed. These two catalyst beds were divided by quartz wool. Prior to testing, the catalyst samples were reduced at 400 °C in 30% H_2_/Ar for 2 h. A mixture of gases was introduced as CH_4_/O_2_/Ar at a molar ratio of 20/10/70% *v*/*v*. The experiments were carried out at 400–650 °C with gas flow rate of 100 mL/min. 

The gas products from the reaction tests (H_2_, CO, CO_2_, O_2_, and CH_4_) were analyzed via an online gas chromatograph (Shimadzu GC-2014, Tokyo, Japan). The mixture gas products were satisfactorily separated by the use of a 60/80 Carboxen-1000 column (15 ft × 1/8 in SS, 2.1 mm ID; Supelco, USA) within the temperature range of 35 °C to 220 °C. The outlet of the gas chromatograph column was directly connected to both the thermal conductivity detector and flame ionization detector. The catalyst activity was defined in terms of the CH_4_ and O_2_ conversions, and product (H_2_, CO, and CO_2_) yields were defined as:

$$\%\;{\mathrm{CH}}_{4}\;\mathrm{Conversion}=\frac{{CH}_{4}\;in\;-{CH}_{4}\;out}{{CH}_{4}\;in}\times 100$$

$$\%\;O_{2}\;\mathrm{Conversion}=\frac{O_{2}\;in\;-\;O_{2}\;out}{O_{2}\;in}\times 100$$

$$\%\;H_{2}\;\mathrm{Yield}=\frac{H_{2}\;out}{{CH}_{4}\;in\times 2}\times 100$$

$$\%\;\mathrm{carbon}\;\mathrm{selectivity}=\frac{CO\;out\;or\;{CO}_{2}\;out}{CO\;out+{CO}_{2}\;out}\times 100$$

### 2.3. Catalytic Partial Oxidation of Methane and Water Gas Shift Reaction

In the study of catalytic partial oxidation of methane, the reaction was carried out in a tubular quartz reactor (OD 3/8 in, length 400 mm). The catalysts were filled in the reactor and reduced to 100 mL/min of H_2_ mixed gas (30% H_2_ in Ar) at 400 °C for 2 h. Afterward, a mixture of 20% CH_4_ and 10% O_2_ in Ar was fed to the reactor at a flow rate of 100 mL/min. The experiments were studied at 400–650 °C. The outlet gas products were heated by heating cable at 150 °C in order to prevent the condensation of water in a tube. Water was removed from gas products by cold trap before being injected into the GC. The catalytic performance was studied in 2 sections;

#### 2.3.1. Single Catalyst

In this section, 5%Ni/GDC, 1%Re-4%Ni/GDC, 5%Cu/GDC, and 1%Re-4%Cu/GDC were studied for catalytic activity in methane partial oxidation reaction and water gas shift reaction. The experiments were performed on powder catalysts with a weight of 100 mg. 

#### 2.3.2. Dual-Bed Catalyst

**Figure 1:**
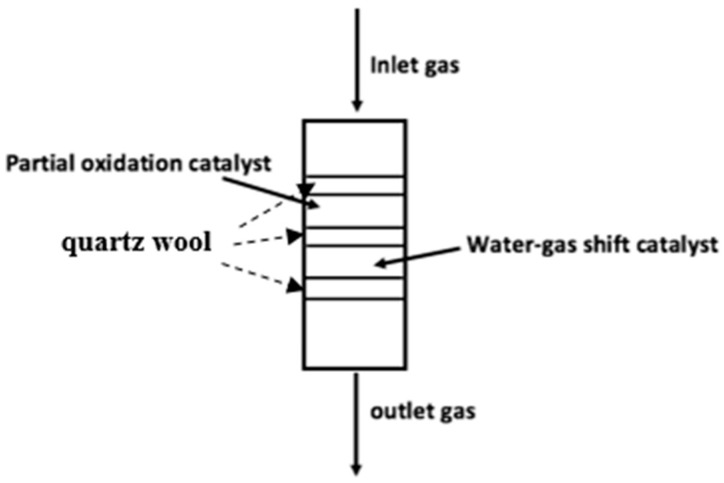
Reactor of the dual-bed catalyst system.

In this section, 100 mg of 1%Re-4%Ni/GDC catalyst was combined with 50 mg of 1%Re-4%Cu/GDC catalyst in the same reactor as shown in Figure 1.

## 3. Results and Discussion

### 3.1. Properties of the Prepared Catalysts

**Figure 2:**
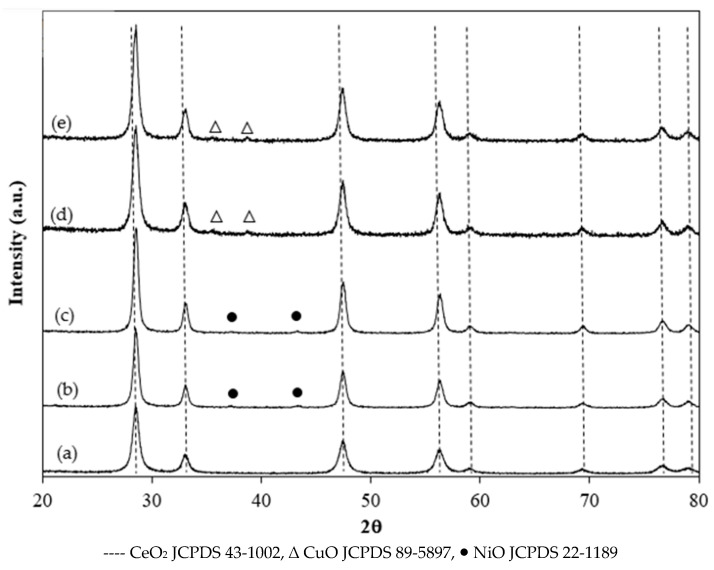
XRD patterns of fresh (**a**) GDC, (**b**) 5%Ni/GDC, (**c**) 1%Re4%Ni/GDC, (**d**) 5%Cu/GDC, and (**e**) 1%Re4%Cu/GDC catalysts.

Figure 2 presents powder X-ray diffraction (XRD) patterns of fresh Ni and Cu catalysts. All catalysts exhibit characteristic XRD peaks for cubic CeO_2_ (JCPDS No. 43-1002). Fresh 5%Ni/GDC and 1%Re4%5Ni/GDC catalysts present NiO peaks at 2θ values of 37.2° and 43.3° whereas very weak CuO peaks at 2θ values of 35.7° and 39.1° can be found in 5%Cu/GDC and 1%Re4%NiCu/GDC catalysts. The diffraction peaks of Ni and Cu catalysts shift to higher diffraction angles compared with the diffraction peaks of GDC support because of the reduction in the cell dimension after calcination at high temperature (650 °C). Due to the nature of the impregnation method, Ni, Cu, and Re can not be incorporated into the ceria lattice, but the lattice contraction is due to the decomposition of surface hydroxyls during calcination. The CeO_2_ crystallite sizes were calculated from X-ray line broadening using the Debye Scherrer equation. Table 1 summarizes the results from BET surface area analysis and crystallite size of GDC support and metals (Ni, Cu, Re-Ni, and Re-Cu) supported on GDC. The BET surface area, pore volume, and pore size of the GDC support was 85.5 m^2^ g^−^^1^, 0.30 cm^3^ g^−^^1^_,_ and 17.6 nm, respectively. Loading of metals onto GDC causes an increase in CeO_2_ crystallite size and a decrease in the BET surface area and total pore volume. This result was considered to be caused by the growth and aggregation of CeO_2_ particles after calcination at 650 °C. In addition, all samples exhibit a mesoporous structure with an average pore size between 17–24 nm. Among the supported metal catalysts, the BET surface area of 1%Re-4%Cu/GDC is higher than that of other catalysts. It can be suggested that Re is a suitable promoter for Cu/GDC because it can help increase surface area, which is one of the factors for improving the catalytic activity of many reactions.

**Figure 3:**
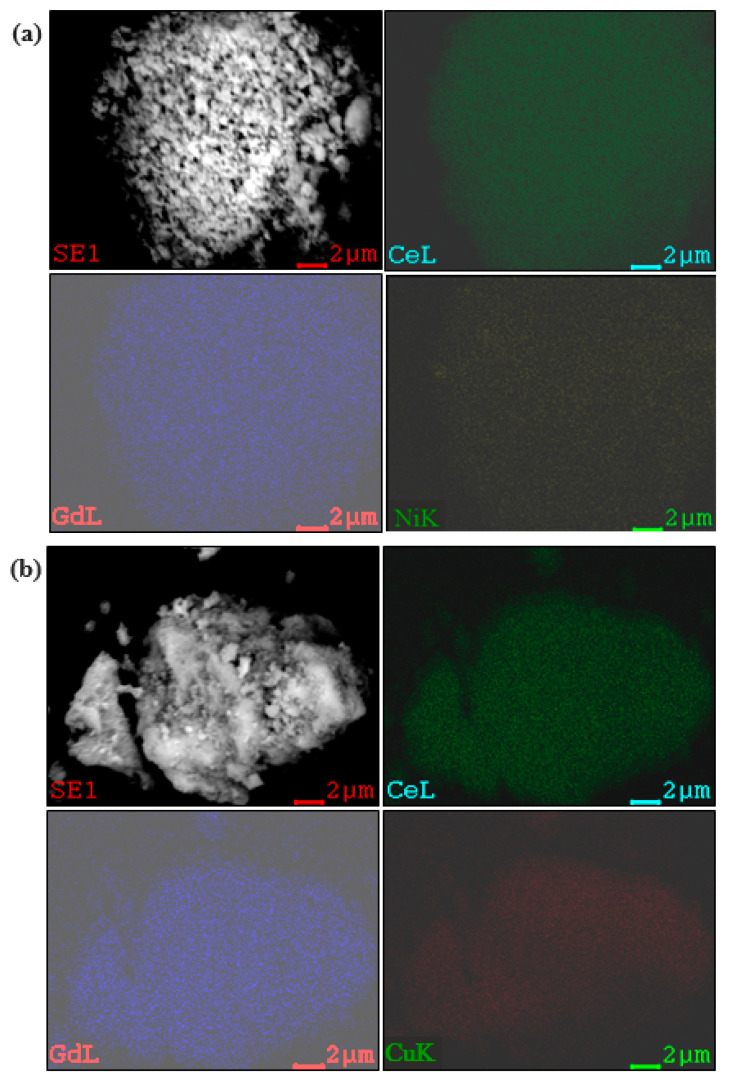
SEM image and elemental mapping of 5%Ni/GDC (**a**) and 5%Cu/GDC (**b**).

Figure 3 shows a Scanning electron microscopy (SEM) image of the fresh 5%Ni/GDC and 5%Cu/GDC catalysts with the corresponding elemental mapping of Cu, Ni, Ce, and Gd. The elemental mapping presents a homogeneous distribution of Cu, Ni, Ce, and Gd species. For the image of the 5%Ni/GDC sample, fewer large particles can be seen than in the image of the 5%Cu/GDC sample.

**Figure 4:**
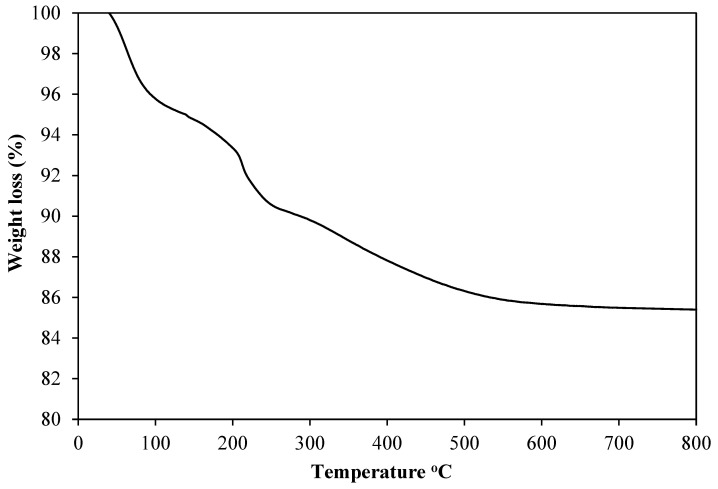
TGA of the uncalcined 1%Re4%Ni/GDC catalyst.

Thermal gravimetric analysis (TGA) was used to investigate the thermal stability of uncalcined 5%Ni/GDC catalyst in the presence of air (Figure 4). Three distinct mass loss regions were found for the uncalcined sample. In the first step, a weight loss of 6.5% at temperature 100–200 °C attributed to loss of adsorbed water. In the second step, a weight loss of 3.2% at 200–300 °C was due to the decomposition of hydroxides releasing water molecules, and the third weight loss of 4.5% at 300–600 °C was attributed to the loss of residual organic materials in the sample [21].

**Figure 5:**
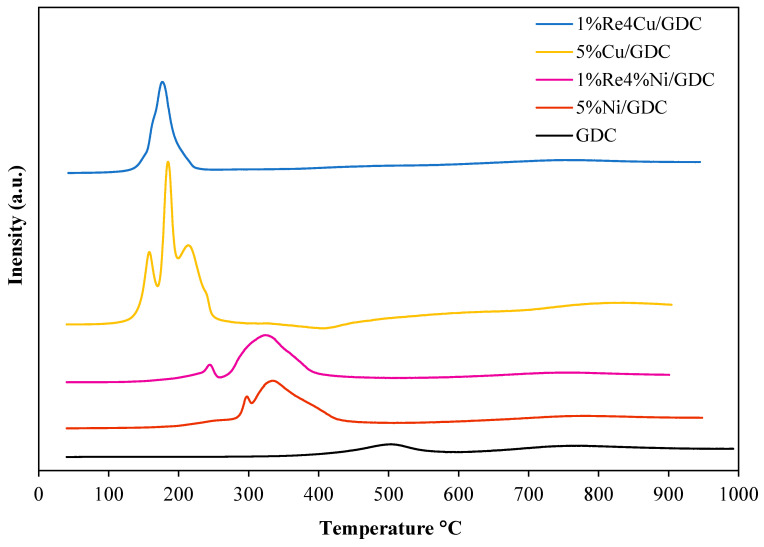
H_2_-TPR profiles of Ni and Cu catalysts.

The H_2_-TPR profiles of supported GDC catalysts are summarized in Figure 5. The H_2_-TPR profile of the 5%Ni/GDC catalyst displayed two reduction peaks. The first reduction peak was located at 300 °C, which corresponded to the reduction of small crystallite NiO, and the second peak at 340 °C corresponded to the reduction of larger crystallite NiO [22,23]. For 1%Re-4%Ni/GDC catalyst, the reduction peak was shifted to a lower temperature. In addition, H_2_-TPR peak areas were an indicator to explain the reducibility of the catalyst; the higher the peak areas indicates the stronger the reducibility of the catalysts. It could be seen that the 1%Re-4%Ni/GDC catalyst illustrated higher TPR peak areas than 5%Ni/GDC catalyst, indicating that the Re addition could improve the catalyst reducibility. For the H_2_-TPR profile of 5%Cu/GDC catalyst, three reduction peaks were observed at 160 °C, 187 °C and 214 °C, which could be assigned to the reduction of small crystallite copper oxide, reduction of copper oxide interacting with surface ceria, and larger crystallite copper oxide, respectively [24]. The H_2_-TPR of 1%Re4%Cu/GDC is different from those of monometallic 5%Cu/GDC catalysts. A concurrent reduction of metal oxide species appears at 170 °C. An electron density transfer between Re, Cu, and GDC may occur, resulting in the reduction of the catalyst surface is easier.

**Figure 6:**
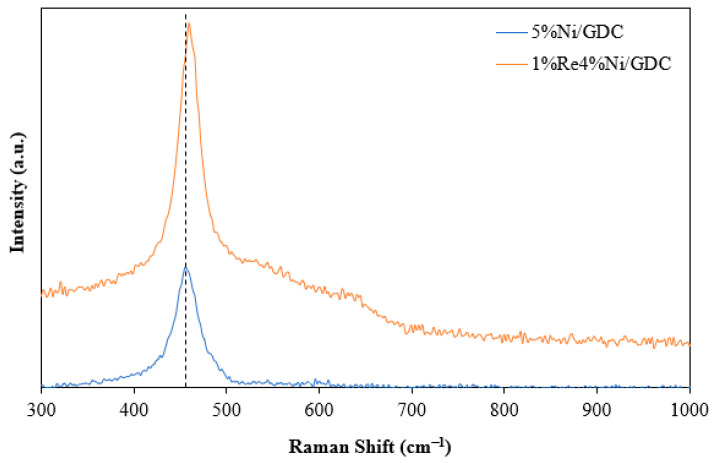
Raman spectra of 5%Ni/GDC and 1%Re4%Ni/GDC catalysts.

Raman spectroscopy was used to investigate oxygen vacancy in the catalyst. Doping ceria by the substitution of two Ce^4+^ ions in the CeO_2_ lattice with Gd^3+^ results in an O ion being eliminated to conserve the charge. The presence of Ce^3+,^ in which the Ce^4+^ ions are replaced by Ce^3+^ ions for oxygen vacancy production, causes the lattice expansion. Oxygen vacancy is related to catalytic performance in many reactions. As can be seen in Figure 6, a Raman peak at around 460 cm^−1^, characteristic of the symmetrical stretching vibration created by eight O atoms bound to one Ce atom, is observed [25]. Furthermore, a red shift of the F_2g_ peak was observed in 1%Re4%Ni/GDC catalyst due to the increased concentration of Ce^3+^ in the CeO_2_-based catalyst. Moreover, other broad peaks in the range of 500–700 cm^−1^ (denoted as D band) are caused by oxygen vacancies in the catalysts [26]. Comparison between 5%Ni/GDC and 1%Re4%Ni/GDC catalysts indicates that the intensities of the D band were increased after Re addition, suggesting that Re promoted the generation of oxygen vacancies in Ni/GDC catalyst.

**Figure 7:**
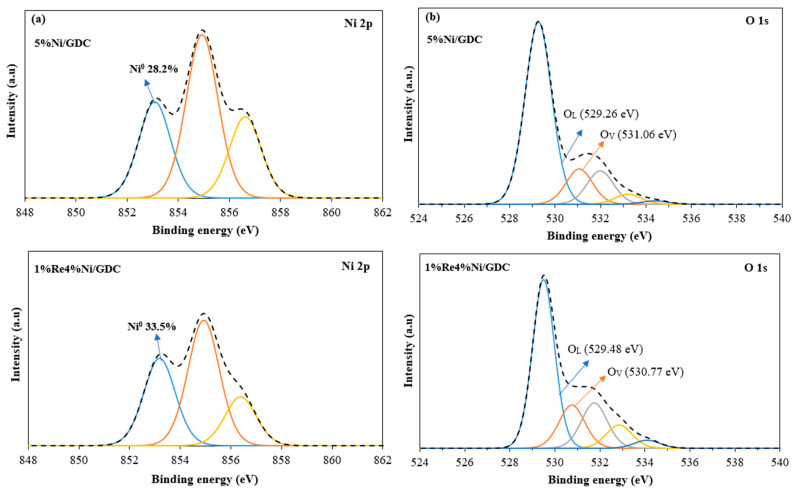
XPS spectra of reduced Ni catalysts before the reaction for Ni 2p_3/2_ (**a**) and O 1s (**b**).

Surface analyses for the Ni and O species present on the catalyst surface were investigated by X-ray photoelectron spectroscopy (XPS). Ni 2p_3/2_ and O 1s XPS spectra of reduced Ni/GDC and ReNi/GDC catalysts were deconvoluted, as shown in Figure 7. Before XPS measurement, the synthesized catalyst was reduced with 5%H_2_/N_2_ at 400 for 1 h. Therefore, nickel was in the metallic state after the reduction. However, the presence of the different Ni species is due to the interaction between Ni and GDC. From Figure 7a, the peak around 853.6–853.8 eV was assigned to Ni^0^, whereas two peaks at higher binding energy were attributed to the Ni 2p_3/2_ of NiO [27]. In addition, It was found that the ratio of Ni^0^/(Ni^0^ + Ni^2+^) of 1%Re4%Ni/GDC (33.5%) was higher than that of 5%Ni/GDC (28.2%). Ni^0^ site is the dominant active site in accelerating the reactants [28]. Therefore, an increase of Ni^0^ amount in 1%Re4%Ni/GDC implies a superior catalytic activity of Ni catalyst with rhenium addition.

The O 1s XPS analysis was performed to confirm the presence of oxygen vacancies in the synthesized catalysts (Figure 7b). The peak ranges from 529.26 to 529.48 eV, 530.77–531.06 eV, and 531.77–534.40 eV represent the lattice oxygen in metal oxide (O_L_), oxygen-deficient regions (O_V_) and chemi-absorbed oxygen (O_C_), respectively [29]. The ratio of O_V_/O_L_ was calculated to illustrate the content of oxygen vacancy on the catalyst surface [30]. These values are 0.30 and 0.19 for 1%Re4%Ni/GDC and 5%Ni/GDC, respectively, indicating that 1%Re4%Ni/GDC has the most abundant oxygen vacancy defects.

In CPOM, Ni^0,^ as the most important active site, can efficiently break the C–H bond of methane, generating hydrogen and surface C species [31,32]. To quantify the number of active sites, CO pulse means were used to measure the adsorbed CO values and calculate the exposed surface area of active Ni^0^ for both Ni/GDC and ReNi/GDC catalysts (Table 2). The Ni^0^ dispersion of ReNi/GDC (6.6%) was higher than that of Ni/GDC (4.1%). Thus, ReNi/GDC obtained a greater exposed surface area of active Ni^0^ (43.5 m^2^/g_Ni_) than that of the Ni/GDC (30 m^2^/g_Ni_), which benefited from high Ni dispersion and reduction. From the results of the chemisorption analysis exhibited in Table 2, it can be seen that Re addition onto Ni/GDC increases Ni surface area and dispersion on the support surface. As expected, the average Ni particle size decreased from 19.4 to 13.8 nm. Generally, catalysts with greater metallic surface area and dispersion provide the enhancement of catalytic performance, as more surface acitve sites are exposed to reactants [22].

### 3.2. Catalytic Performance

**Figure 8:**
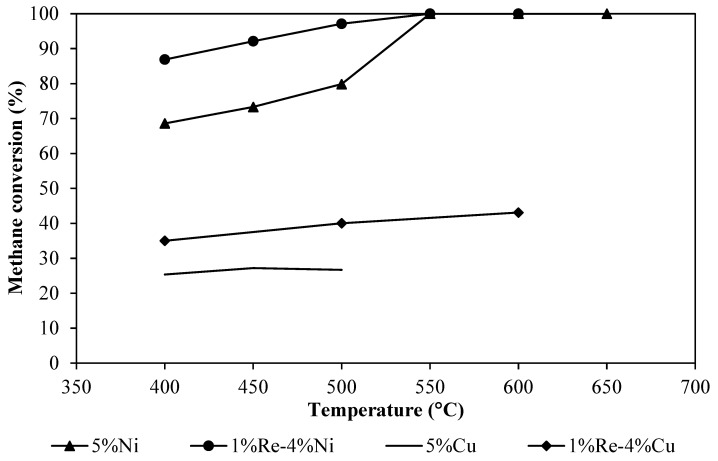
CH_4_ conversion of single catalyst.

The results of CH_4_ partial oxidation using a single catalyst (5%Ni/GDC, 1%Re-4%Ni/GDC, 5%Cu/GDC, and 1%Re-4%Cu/GDC) in terms of CH_4_ and O_2_ conversion, H_2_ yield as well as carbon selectivity were illustrated in Table 3 and Figure 8. The experiments were performed on powder catalysts with a weight of 100 mg. It can be seen that the 5%Ni/GDC catalyst showed good catalytic activity in terms of CH_4_ conversion and H_2_ yield. The catalytic reaction of the 5%Ni/GDC catalyst occurred at 400 °C and was enhanced with an increase in reaction temperature. The highest performance was achieved at 550 °C with methane conversion and hydrogen yield of 100% and 68.85%, respectively. The results indicate that methane was effectively dissociated to generate C species, which is confirmed by the effluents CO and CO_2_. Considering 5%Ni/GDC and 1%Re-4%Ni/GDC catalysts, these catalysts showed higher CH_4_ dissociation activity than Cu catalysts. It strongly indicates that methane dissociation occurs on the metallic Ni active sites. However, a large amount of H_2_ and CO was detected over 1%Re-4%Ni/GDC catalyst. It suggests that the mobile oxygen species in this catalyst play a very important role in CH_4_ conversion and CO production. The O_2_ conversion was found to be 100% for all tests because the produced H_2_ and CO were reacted with the remaining O_2_ and converted into CO_2_ and H_2_O. Comparing this result with the literature data [23,29], it was found that Ni/GDC catalyst showed higher CH_4_ conversion than Ni/CeO_2_ catalyst. The addition of Gd into the CeO_2_ structure improved the catalytic performance in the partial oxidation of CH_4_ because its oxygen storage capacity was increased [33]. Furthermore, an increase in methane conversion may occur from H_2_O and CO_2_ reforming because H_2_O and CO_2_ can be easily dissociated over Ni/GDC catalyst [34,35]. 

From previous studies, methane partial oxidation of Ni/CeO_2_, Ni/ZrO_2,_ and Ni/Ce–ZrO_2_ catalysts was investigated by simultaneous pulse reaction of CH_4_/O_2_ at 600 and 800 °C [36]. When the CH_4_/O_2_ pulse reaction was performed at 600 °C, all the catalysts exhibited similar CH_4_ conversions at about 60%. Ni/CeO_2_ presented 56.5% CO selectivity and 22.7% CO_2_ selectivity, indicating some carbon species deposited on the catalyst surface because of the lack of surface reactive oxygen species. On the other hand, the Ni/Ce–ZrO_2_ catalyst exhibited good performance with approximately 90% of CH_4_ conversion and 95% of CO selectivity at 800 °C. The addition of CeO_2_ to ZrO_2_ increases methane dissociation and oxygen storage capacity in the Ni/Ce–ZrO_2_ catalyst, resulting in reduced carbon deposition on this catalyst. The direct partial oxidation mechanism involves CH_4_ pyrolysis on the surface to produce C and H, followed by the oxidation of C to CO [5,36]. Some recent studies contribute to this mechanism, and Rh and Pt catalysts were investigated for POM [37]. The researchers proposed that the mechanism involved the pyrolysis of methane on the catalyst surface to give surface C and H species. The H atoms dimerize and desorb as H_2_, whereas the surface carbon species react with adsorbed O atoms and desorb as CO. As above, Hu and Ruckenstein [38] suggested that methane and O_2_ react in the adsorbed states through the pyrolysis mechanism over the Ni/SiO_2_ catalyst. They found that the reaction between the surface C and O species was the rate-controlling step.

Therefore, the excellent performance of 1%Re-4%Ni/GDC catalyst for POM in this work was due to Ni active sites and adsorbed oxygen species on the catalyst surface. The good ability to store, release, and transfer O species of ceria-based materials leads to an increased ability to eliminate carbon which would accumulate on the catalyst surface during the decomposition of methane by producing CO and CO_2_. Comparing the results of the partial oxidation of methane at different temperatures, CO_2_ selectivity over all the Ni catalysts declined with enhancing temperature. CO and CO_2_ selectivities in the partial oxidation of methane are governed by the relative carbon amount to oxygen species on the catalyst surface. CH_4_ and O adsorb competitively on metallic Ni. The CH_4_ adsorption rate on nickel can be accelerated by rising temperature while the surface coverage of adsorbed oxygen species decreases with increasing temperature, hence leading to the reduction of CO_2_ selectivity. On the other hand, CO_2_ production and CO selectivity depend on the two competition steps, namely the oxidation and the desorption of CO. The enhancement of reaction temperature favors CO desorption over oxidation, resulting in the increase of CO selectivity [39].

**Figure 9:**
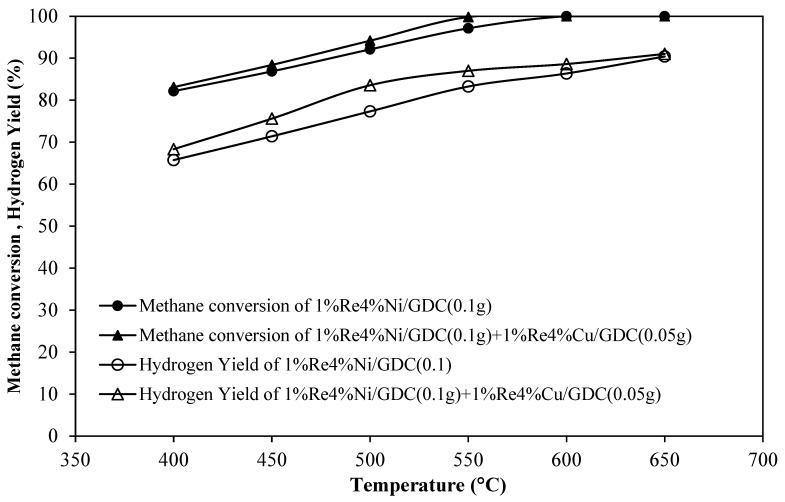
CH_4_ conversion and H_2_ Yield using a dual-bed catalyst compared with a single-bed catalyst of 1%Re4%Ni/GDC catalyst.

To maximizing H_2_ production, a dual-bed catalyst system was studied in this section, 100 mg of nickel-based catalysts (1%Re-4%Ni) were combined with 50 mg of 1%Re-4%Cu/GDC catalyst in the same reactor. From the literature review, Tepamatr et al. (2016) have reported that the Cu/GDC catalyst presented good catalytic activity toward the WGS reaction [14]. Therefore, in this work, Cu/GDC catalysts were used to enhance H_2_ yield in CH_4_ partial oxidation using a dual-bed system. Table 4 and Figure 9 summarize the results of the dual-bed catalyst, which consists of the first POM catalyst layer (Bed 1) and the second WGS catalyst layer (Bed 2). The decreased CO production of the dual-bed catalyst system was due to CO was changed to CO_2_ through the WGS reaction with 1%Re4%Cu/GDC catalyst (Bed 2). In addition, the results showed that the dual-bed system in case of 0.1 g of 1%Re4%Ni/GDC (Bed 1) and 0.05 g of 1%Re4%Cu/GDC (Bed 2) presented H_2_ yield much higher than single bed catalyst at low temperature (400−550 °C) because WGS is an exothermic reaction, thereby the equilibrium is favored by low temperatures. 

In the previous study [15], the dual-bed system (Pt/Ni catalyst) was compared to single monolith platinum and rhodium catalysts for hydrogen production from the auto-thermal partial oxidation of methane. The Pt/Ni catalyst achieved an H_2_ yield of 86% compared to the 88% H_2_ yield of the Rh catalyst. The Pt/Ni catalyst performed as well as the Rh catalyst over a wide range of operating conditions, but the price costs of the Rh catalyst, including blank monolith, are higher than that of the Pt/Ni catalyst by about 40%. In this work, non-noble metal-based catalysts were investigated to reduce the price costs of the catalyst. The dual-bed catalyst showed the best performance, reaching a CH_4_ conversion of 100% and H_2_ yield of 91% at 650 °C. Therefore, a dual-bed bimetallic catalyst composed of 1%Re4%Ni/GDC catalyst followed by a water gas shift catalyst (1%Re4%Cu/GDC) demonstrates comparable CH_4_ conversion and H_2_ yield compared to the best available catalysts in the literature.

## 4. Conclusions

Ni-based catalysts were more active than Cu-based catalysts for partial oxidation of CH_4_. An addition of rhenium caused an increase in catalytic activity and H_2_ yield. A dual-bed catalyst system, by combining the POM catalyst and WGS catalyst, could enhance the H2 yield from the WGS reaction. The best catalytic activity was achieved using a dual-bed catalyst system from the combination of 1%Re4%Ni/GDC (0.1 g) as a partial oxidation catalyst and 1%Re4%Cu/GDC (0.05 g) as a water-gas-shift catalyst. The highest performance was reached at 650 °C with CH_4_ conversion and H_2_ yield of 100% and 91.04%, respectively. From H_2_-TPR analysis, bimetallic catalysts (ReNi, ReCu) illustrated higher TPR peak areas than monometallic catalysts, indicating that Re addition could improve the catalyst reducibility. Furthermore, 1%Re4%Ni/GDC catalyst with a higher Ni surface area provides an increase in catalytic performance as more surface active sites are exposed to reactants.

## Figures and Tables

**Table 1:** BET surface area, pore volume, pore diameter, and crystallite size of GDC support and prepared catalysts.

Catalysts	BET Surface Area ^a^ (m^2^ g^−1^)	Total Pore Volume ^a^ (cm^3^ g^−1^)	Pore Diameter ^a^ (nm)	Crystallite Size ^c^ (nm)
GDC (Daiichi)	85.5	0.30	17.6	12.3
5%Ni/GDC	40.2	0.24	23.9	14.8
5%Cu/GDC	45.6	0.26	22.4	14.2
1%Re4%Ni/GDC	39.6	0.21	21.3	13.2
1%Re4%Cu/GDC	53.2	0.23	17.5	12.8

^a^ Estimated from N_2_ adsorption at −196 °C, ^c^ calculated from the 111 diffraction peak broadening.

**Table 2:** Ni surface area and dispersion of 5%Ni/GDC and 1%Re4%Ni/GDC catalysts estimated from CO-chemisorption.

Catalysts	% Ni Dispersion	Ni Surface Area (m^2^/g_Ni_)	Average Particle Size (nm)
5%Ni/GDC	4.1	30.0	19.4
1%Re4%Ni/GDC	6.6	43.5	13.8

**Table 3:** Catalytic partial oxidation of CH_4_ using a single catalyst.

Catalyst	Temperature (°C)	Conversion (%)		H_2_ Yield (%)	Carbon Selectivity (%)	
		CH_4_	O_2_		CO	CO_2_
5%Ni/GDC (0.1 g)	400	68.60	100.00	53.36	47.78	52.22
	450	73.30	100.00	57.42	48.98	51.02
	500	79.84	100.00	62.79	58.08	41.92
	550	100.00	100.00	68.85	61.42	38.58
	600	100.00	100.00	67.56	61.49	38.51
	650	100.00	100.00	67.64	61.42	38.58
1%Re- 4%Ni/GDC (0.1 g)	400	82.15	100.00	65.74	57.39	42.61
	450	86.88	100.00	71.42	63.97	36.03
	500	92.11	100.00	77.33	69.76	30.24
	550	97.13	100.00	83.26	75.31	24.69
	600	100.00	100.00	86.38	79.05	20.95
	650	100.00	100.00	90.42	82.33	17.67
5%Cu/GDC (0.1 g)	400	25.38	100.00	0.97	-	100
	450	27.21	100.00	0.96	-	100
	500	26.7	100.00	1.10	-	100
1%Re- 4%Cu/GDC (0.1 g)	400	35.01	100.00	1.68	-	100
	500	40.02	100.00	2.71	-	100
	600	43.10	100.00	4.16	-	100

**Table 4:** Catalytic partial oxidation of CH_4_ using a dual-bed catalyst system of 0.1 g of 1%Re4%Ni/GDC (Bed 1) and 0.05 g of 1%Re4%Cu/GDC (Bed 2).

Temperature (°C)	Conversion (%)		H_2_ Yield (%)	Carbon Selectivity (%)	
	CH_4_	O_2_		CO	CO_2_
400	83.12	100.00	68.34	48.02	51.98
450	88.41	100.00	75.61	52.85	47.15
500	94.17	100.00	83.57	59.45	40.55
550	99.81	100.00	87.00	65.38	34.62
600	100.00	100.00	88.63	68.83	31.17
650	100.00	100.00	91.04	70.14	29.86

## Data Availability

Data are contained within the article.

## References

[1] Staffell I., Scamman D., Abad A.V., Balcombe P., Dodds P.E., Ekins P., Shahd N., Ward K.R. (2019). The role of hydrogen and fuel cells in the global energy system. Energy Environ. Sci..

[2] Ail S.S., Dasappa S. (2016). Biomass to liquid transportation fuel via Fischer Tropsch synthesis—Technology review and current scenario. Renew. Sustain. Energy Rev..

[3] Park T.Y., Nam I.S., Kim Y.G. (1997). Kinetic Analysis of Mixed Alcohol Synthesis from Syngas over K/MoS_2_ Catalyst. Ind. Eng. Chem. Res..

[4] Enger B.C., Lødeng R., Holmen A. (2008). A review of catalytic partial oxidation of methane to synthesis gas with emphasis on reaction mechanisms over transition metal catalysts. Appl. Catal. A Gen..

[5] York A.P.E., Xiao T., Green M.L.H. (2003). Brief overview of the partial oxidation of methane to synthesis gas. Top. Catal..

[6] Singha R.K., Ghosh S., Acharyya S.S., Yadav A., Shukla A., Sasaki T., Venezia A.M., Chandrashekar P., Rajaram B. (2016). Partial oxidation of methane to synthesis gas over Pt nanoparticles supported on nanocrystalline CeO_2_ catalyst. Catal. Sci. Technol..

[7] Cheephat C., Daorattanachai P., Devahastin S., Laosiripojana N. (2018). Partial oxidation of methane over monometallic and bimetallic Ni-, Rh-, Rebased catalysts: Effects of Re addition, co-fed reactants and catalyst support. Appl. Catal. A Gen..

[8] Ouyang M., Boldrin P., Maher R.C., Chen X., Liu X., Cohen L.F., Brandon N.P. (2019). A mechanistic study of the interactions between methane and nickel supported on doped ceria. Appl. Catal. B Environ..

[9] Tepamatr P., Buarod E., Laosiripojana N., Charojrochkul S. (2015). Study of water gas shift reaction over ceria based catalysts in solid oxide fuel cells. ECS Trans..

[10] Ferreira A.C., Ferraria A.M., do Rego A.M.B., Gonçalves A.P., Girão A.V., Correia R., Gasche T.A., Branco J.B. (2010). Partial oxidation of methane over bimetallic copper–cerium oxide catalysts. J. Mol. Catal. A Chem..

[11] Beckers J., Rothenberg G. (2008). Redox properties of doped and supported copper–ceria catalysts. Dalton Trans..

[12] Lomonaco J.G., Tojira O., Charojrochkul S., Tepamatr P. (2022). Structure-activity relationship of ceria based catalyst for hydrogen production. Chiang Mai J. Sci..

[13] Meng F., Li X., Lv X., Li Z. (2018). CO hydrogenation combined with water-gas-shift reaction for synthetic natural gas production: A thermodynamic and experimental study. Int. J. Coal Sci. Technol..

[14] Tepamatr P., Laosiripojana N., Charojrochkul S. (2016). Water gas shift reaction over monometallic and bimetallic catalysts supported by mixed oxide materials. Appl. Catal. A Gen..

[15] Chen W.H., Lin M.R., Lu J.J., Chao Y., Leu T.S. (2010). Thermodynamic analysis of hydrogen production from methane via autothermal reforming and partial oxidation followed by water gas shift reaction. Int. J. Hydrogen Energy.

[16] Tong G.C.M., Flynn J., Leclerc C.A. (2005). A dual catalyst bed for the autothermal partial oxidation of methane to synthesis gas. Catal. Lett..

[17] Batista M., Assaf E.M., Assaf J.M., Ticianelli E.A. (2006). Double bed reactor for the simultaneous steam reforming of ethanol and water gas shift reactions. Int. J. Hydrogen Energy.

[18] Zhu H., Gou L., Li C., Fu X., Weng Y., Chen L., Fang B., Shuai L., Liao G. (2024). Dual interfacial electric fields in black phosphorus/MXene/MBene enhance broad-spectrum carrier migration efficiency of photocatalytic devices. Device.

[19] Wang Z., Ding G., Zhang J., Lv X., Wang P., Shuai L., Li C., Ni Y., Liao G. (2024). Critical role of hydrogen bonding between microcrystalline cellulose and g-C_3_N_4_ enables highly efficient photocatalysis. Chem. Commun..

[20] Maiya P.S., Anderson T.J., Mieville R.L., Dusek J.T., Picciolo J.J., Balachandran U. (2000). Maximizing H_2_ production by combined partial oxidation of CH_4_ and water gas shift reaction. Appl. Catal. A Gen..

[21] Acharyya S.S., Ghosh S., Adak S., Singh R., Saran S., Bal R. (2015). Selective Oxidation of n-Hexane by Cu (II) Nanoclusters Supported on Nanocrystalline Zirconia Catalyst. J. Nanosci. Nanotechnol..

[22] Lomonaco J.G., Sesuk T., Charojrochkul S., Tepamatr P. (2023). Effect of Re Addition on the Water–Gas Shift Activity of Ni Catalyst Supported by Mixed Oxide Materials for H_2_ Production. Catalysts.

[23] Xu S., Wang X. (2004). Highly active and coking resistant Ni/CeO_2_-ZrO_2_ catalyst for partial oxidation of methane. Fuel.

[24] Dow W.P., Wang Y.P., Huang T.J. (2000). TPR and XRD studies of yttria-doped ceria/γ-alumina-supported copper oxide catalyst. Appl. Catal. A Gen..

[25] Rui N., Zhang X., Zhang F., Liu Z., Cao X., Xie Z., Zou R., Senanayake S.D., Yang Y., Rodriguez J.A. (2021). Highly active Ni/CeO_2_ catalyst for CO_2_ methanation: Preparation and characterization. Appl. Catal. B Environ..

[26] Guo D., Lu Y., Ruan Y., Zhao Y., Zhao Y., Wang S., Ma X. (2020). Effects of extrinsic defects originating from the interfacial reaction of CeO_2-x_-nickel silicate on catalytic performance in methane dry reforming. Appl. Catal. B Environ..

[27] Bortolozzi J.P., Weiss T., Gutierrez L.B., Ulla M.A. (2014). Comparison of Ni and Ni-Ce/Al_2_O_3_ catalysts in granulated and structured forms: Their possible use in the oxidative dehydrogenation of ethane reaction. Chem. Eng. J..

[28] Liu L., Wang Q., Song J., Ahmad S., Yang X., Sun Y. (2017). Plasma-assisted catalytic reforming of toluene to hydrogen rich syngas. Catal. Sci. Technol..

[29] Kumar V.P., Pradeep C., Rajsha M.M., Rishad K.P.M., Radhakrishnan P., Mujeeb A. (2023). Band-gap dependence of two-photon absorption mechanism in NiO nanoparticles synthesized at different calcination temperatures. Opt. Mater..

[30] Ansari S.A., Khan M.M., Ansari M.O., Kalathil S., Lee J., Cho M.H. (2014). Band gap engineering of CeO_2_ nanostructure using an electrochemically active biofilm for visible light applications. RSC Adv..

[31] Wang H.T., Li Z.H., Tian S.X. (2004). Effect of promoters on the catalytic performance of Ni/Al_2_O_3_ catalyst for partial oxidation of methane to syngas. React. Kinet. Catal. Lett..

[32] Singha R.K., Shukla A., Yadav A., Konathala L.N.S., Bal R. (2017). Effect of metal-support interaction on activity and stability of Ni-CeO_2_ catalyst for partial oxidation of methane. Appl. Catal. B.

[33] Salazar-Villalpando M.D., Reyes B. (2009). Hydrogen production over Ni/ceria-supported catalysts by partial oxidation of methane. Int. J. Hydrogen Energy.

[34] Huang T.J., Yu T.C. (2005). Effect of steam and carbon dioxide pretreatments on methane decomposition and carbon gasification over doped-ceria supported nickel catalyst. Catal. Lett..

[35] Liu K., Liao Y., Wang P., Fang X., Zhu J., Liao G., Xu X. (2024). Lattice capacity-dependent activity for CO_2_ methanation: Crafting Ni/CeO_2_ catalysts with outstanding performance at low temperatures. Nanoscale.

[36] Dong W.S., Jun K.W., Roh H.S., Liu Z.W., Park S.E. (2002). Comparative study on partial oxidation of methane over Ni/ZrO_2_, Ni/CeO_2_ and Ni/Ce–ZrO_2_ catalysts. Catal. Lett..

[37] Hickman D.A., Schmidt L.D. (1993). Production of Syngas by Direct Catalytic Oxidation of Methane. Science.

[38] Hu Y.H., Ruckenstein E. (2004). Catalytic Conversion of Methane to Synthesis Gas by Partial Oxidation and CO_2_ Reforming. Adv. Catal..

[39] Liao M.S., Au C.T., Ng C.F. (1997). Methane dissociation on Ni, Pd, Pt and Cu metal (111) surfaces—A theoretical comparative study. Chem. Phys. Lett..

